# Rapid growth in the COVID-19 era

**DOI:** 10.1557/s43577-021-00185-2

**Published:** 2021-09-29

**Authors:** Yerim Lee, Michelle Ng, Kristin Daniel, Elizabeth Wayne

**Affiliations:** Department of Biomedical Engineering, and Department of Chemical Engineering, Carnegie Mellon University, Pittsburgh, PA USA

**Keywords:** Rapid-antigen detection, Therapeutic antibodies, Drug repurposing, mRNA lipid nanoparticle vaccine, Operation WARP speed

## Abstract

**Abstract:**

From Operation Warp Speed to the lipid mRNA vaccine, the COVID-19 pandemic has been a watershed moment for technological development, production, and implementation. The scale and pace of innovation and global collaboration has likely not been experienced since World War II. This article highlights some of the engineering accomplishments that occurred during the pandemic. We provide a broad overview of the technological achievements in vaccine design, antibody engineering, drug repurposing, and rapid diagnostic testing. We also discuss what the future of these technologies and the future of large-scale collaborations might look like moving forward.

**Graphic abstract:**

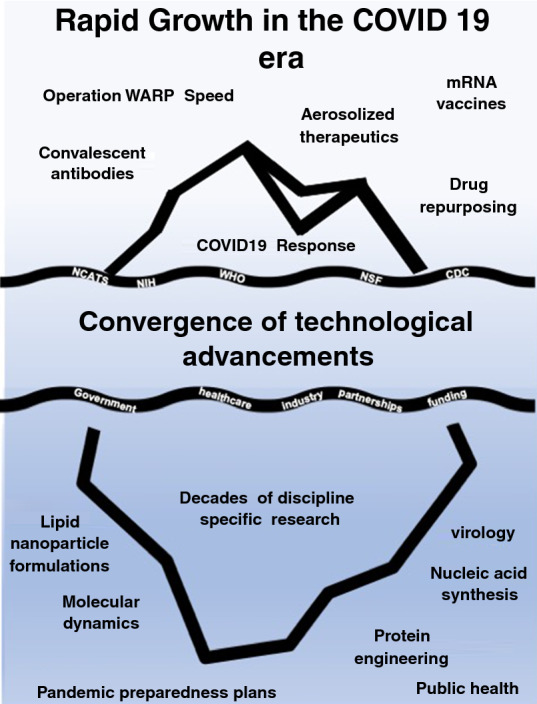

## Introduction

The urgent diagnostic, therapeutic, and protective equipment needs posed by the COVID-19 pandemic ushered in a wave of global collaboration that arguably has not been experienced since World War II. The contributions of scientists and engineers shaped the style of warfare and fundamentally changed the way that scientists, scientific institutions, and governments interacted.^1^ Many of the technological developments are so common now that they seem trivial such as the advancement of electrical circuits, oscilloscopes, and microwave generators, which were used for radar surveillance and making computers.^2,3^ Others had long-term but more complicated impact such as the formation of the Manhattan Project and the development of the nuclear bomb.^4^ However, one of the most pivotal collaborations was a medical undertaking. It is here that we can draw the most meaningful comparisons to grasp the magnitude of growth that occurred during COVID-19.

Many soldiers died from post wound infections such as gangrene. An estimated 15% of wounded soldiers died from infections in World War I compared to 3% in World War II.^5^ The difference was the mass production of penicillin. Alexander Fleming haphazardly discovered penicillin in 1925 as a mold growing in an overlooked petri dish.^6,7^ It would take more than a decade before those results were revisited because of the difficulty in purification. In 1939, a group of British chemists led by H. Florey were able to purify penicillin and published seminal papers demonstrating its efficacy in an animal model the following year.^6,8^ After this monumental publication, a flurry of clinical trials began. Doctors used purified penicillin to treat patients who had shown no efficacy with the existing treatment.^8^ Unfortunately, while there were some successes, many patients still succumbed to infection due to inefficient penicillin doses. Penicillin could be purified and concentrated, but it was still difficult to achieve sufficient amounts needed to treat a single patient, let alone an entire population. At this point, Florey began pursuing avenues to produce larger quantities of penicillin. This led him to collaborations with the US Department of Agriculture and pharmaceutical companies such as Merck, Pfizer, Abbot, and what is now Bristol Myers Squibb.^6^ The interactive collaboration led to fermentation research that increased penicillin yield, new batch reactors that facilitate high-volume production. Combined with the government financial backing from the US War Production Board,^9^ penicillin production went from enough to treat a single patient in 1942 to being able to deliver 2.5 million doses before the Battle of Normandy in 1944.^10^ Simultaneously, other countries began to increase their own production of penicillin. In this manner, the success of penicillin has direct connections with the speedy production and distribution of the mRNA vaccine.

Although the COVID-19 pandemic was not a global political war, it was and remains a global biological challenge that demanded alliances and participation between academic and clinical medicine, industry, and government. This article will give an overview of the technological victors of the COVID-19 pandemic. The mRNA vaccine, SARS-CoV-2 antibody development, rapid testing diagnostics, and personal protective equipment (PPE) manufacturing were all critical to curbing the COVID-19 death toll. In addition, the article will highlight the materials research innovations that underpinned these developments.

## mRNA-LNP vaccine development

The respiratory, highly infectious nature of the SARS-CoV-2 coronavirus necessitated the rapid, safe, and efficacious vaccine development. Dozens of biotechnology companies leapt into action as soon as the SARS-CoV-2 genome was publicly released, assisted by the accelerated clinical trial and approval process implemented by worldwide regulatory bodies. As of March 25, 2021, there were 13 vaccines across the world that had been approved for human use and 58 in development.^11^ Of these, mRNA vaccines have received widespread attention both for being the fastest to market and the first mRNA-based vaccines approved for use in humans (**Table ****I**).

**Table I:** Currently researched mRNA vaccines by company and country.

Vaccine Name	Company	Country of Origin	Phase	References
Comirnaty (BNT162b2)	Pfizer, BioNTech	US, Germany, China	Approved	22, 23
mRNA-1273	Moderna	US	Approved	24, 25
CVnCov	CureVac, GSK	Germany, UK, Belgium	2b/3	26
BNT162^a^	Pfizer, BioNTech	US, Germany	1/2/3	
MRT5500	Sanofi, Translate Bio	France, US	1/2	29
PTX-COVID19-B	Providence Therapeutics	Canada	1	30
No name announced	Chulalongkorn University	Thailand	Preclinical	31

Concurrent clinical trial phases also shown. Approved for use based on FDA emergency use authorization. As of March 25, 2021.

^a^Vaccine series for several COVID-19 variants, not including BNT162b2.

mRNA vaccine technology existed long before COVID-19 but remained a scientific prospect due to the formulation challenges and poor success in clinical trials.^12^ A successful mRNA delivery vector must avoid the immune system’s ability to recognize and quickly destroy foreign nucleic acids, but also easily penetrate cells’ lipid membrane barrier to deliver the mRNA into the cytoplasm. Lipid nanoparticles (LNPs) addressed mRNA enzymatic degradation and low cell uptake, some of the biggest challenges in developing mRNA vaccines.^12^ The two mRNA vaccines currently approved for public use are mRNA-lipid nanoparticle (mRNA-LNP) vaccines that encode for the SARS-CoV-2 spike protein.^13^

Instead, research and funding had focused on other vaccine platforms that had proven safety and efficacy, including live attenuated viruses, recombinant proteins, or viral vectors. The COVID-19 pandemic led to significant urgency that pushed the innovation of mRNA vaccines forward, with lasting implications on our ability to fight future pandemics. Prior to mRNA-LNP vaccines, there was only one FDA-approved nucleic acid-LNP therapeutic, Alnylam’s Patisiran.^13^ Since that drug’s launch in 2018, LNP delivery systems had fallen out of favor because of their side effects. The successful use of LNPs in SARS-CoV-2 vaccines has cast a renewed global spotlight on LNPs.

The speed of mRNA vaccine development was first and foremost made possible by the previous research done with MERS-CoV and SARS-CoV. In developing vaccines for these viruses, stabilizing mutations were introduced to the mRNA, increasing the vaccine’s antibody recognition and immunogenicity.^14^ Because this modification was already shown to work in other coronaviruses, scientists had an existing reference point when creating a stable spike protein for the SARS-CoV-2 vaccine. Prototype pathogens, in this case MERS-CoV and SARS-CoV, can be used to optimize vaccine designs for different viral families in anticipation of outbreaks.^15^ Previous research done with the prototype pathogen helped shorten the time span of vaccine development for SARS-CoV-2 by several months.

Using LNPs as the mRNA delivery platform also has additional benefits. Cellular uptake of mRNA-LNPs can vary by route of administration. In comparison to intravenous routes, mRNA-LNPS delivery via intramuscular and intradermal routes have exhibited higher protein expression.^15^ If the protein is an antigen, then sustained antigen availability can correlate with the intensity, duration, and cellular composition of the immune response.^16^ LNPs can also possess immunostimulatory properties, inducing immune responses comparable to other traditional vaccine adjuvants like Alum.^12^ The LNP platform is highly compatible with nucleic acid formulations, which made it readily available for incorporation with the SARS-CoV-2 mRNA sequence. In contrast, traditional vaccines require extensive manufacture of cell lines to produce attenuated viruses or clinical-grade protein subunits. As a result, mRNA-LNP vaccines are also more cost effective and scalable for the needs of a global pandemic. Further, mRNA-LNP vaccines are translated within the cell cytoplasm and as such has no risk of integrating into host chromosomes, unlike DNA-platform vaccines.^17^

The “plug and play” nature of the mRNA-LNP vaccine platform has reduced the barrier to new or booster vaccine development timelines. Nonetheless, emerging SARS-CoV-2 variants have raised concerns about the effectiveness of previously developed and administered vaccines. These data are likely to change as the pandemic evolves; however, currently published clinical studies have demonstrated that mRNA-LNP vaccines elicit strong neutralizing antibody and CD4 + T cell responses are predicted predicted to help mount an effective immune response against SARS-CoV-2 as well as its variants.^18^ Other clinical studies have shown that the vaccine-elicited antibodies may be less effective at neutralizing variants with certain spike mutations. Pfizer-BioNTech’s as well as its competitors have begun to explore the significance of booster doses against SARS-CoV-2 variants, though as of the writing of this article, the data have not been published.^19^

## Antibody development

Since vaccine-mediated adaptive immune responses take weeks to develop, individuals with high risks of contracting the virus may benefit from an immediate immunity obtained through passive antibody therapy.^20,21^ Antibodies treat infection via neutralization, opsonization, and complement activation. COVID-19 antibodies target the SARS-CoV-2 spike (S) protein because it is heavily involved in virus entry and infection of cells and transmission.^22^ Because mortality and complications seem to be associated with high viral loads, treatment with neutralizing antibodies may decrease the severity of the condition.^23^ Current methods include convalescent and monoclonal antibodies and, overall, studies suggest that this method of treatment is most effective for individuals with mild to moderate forms of the disease and are in the early stages of infection.^24,25^ Clinical trials with larger sample sizes are ongoing to continue examining their safety and efficacy in different groups with varying risk factors and stages of infection.

## Convalescent antibodies

Convalescent antibodies are obtained from donors who recovered from infection with SARS-CoV-2. Convalescent antibodies have been used for treating infectious diseases when there is no specific treatment.^21^ However, there is a lack of wide-scale randomized testing to assess the effectiveness of convalescent plasma therapy as a standard treatment for COVID-19, although trends in various clinical assessments suggest that they can be beneficial especially for those in early stages of infection.^25^ Challenges in convalescent plasma therapy include difficulties in collection and selection: plasma needs to be collected from donors and then analyzed for high IgG levels. Also, plasma may be contaminated with infectious agents and there are also risks associated with transfusion including circulatory system overload.^20^ However, analysis of convalescent plasma can be used to identify potential antibodies for monoclonal antibodies therapy development.^20^

## Monoclonal antibodies

The drugs Casirivimab and Imdevimab contain REGN-COV2 antibody cocktails that were effective in reducing viral load, especially in patients with high baseline viral load or those whose own adaptive immune response had not been initiated.^23^ Similarly, drugs Bamlanivimab and Etesevimab containing neutralizing antibody LY-CoV555 administered together helped to reduce mortality for those without hospitalizations.^26^ Casirivimab and Imdevimab are co-administered intravenously and can currently be used in patients with mild to moderate COVID-19 (starting from age 12 and more than 40 kg) who have risks of progressing into severe forms. Similar regulations for Bamlanivimab and Etesevimab exist;^27^ use of these drugs are prohibited for those with severe COVID-19 because observations suggest that complications may arise that cause the need for high oxygen flow or mechanical ventilation.

## Aerosolized antibodies

A less obvious winner in the COVID-19 therapeutics race is the advancement of aerosolized therapeutics, engineered particles that can engage with SARS-CoV-2 virus while in air circulation. Nebulizing monoclonal antibodies to facilitate lung delivery was growing in popularity, but probably accelerated during the COVID-19 pandemic.^28^ Several strategies attempt to neutralize virus particles to reduce the risk of SARS-CoV-2 transmission. One such example is Engineering Water Nanostructures (EWNS), nanoparticle structs that contain reactive oxygen species that an deactivate airborne virus and/or microbes.^29,30^

Inhalation was used as a therapeutic delivery route. The transition from intravenous to inhalation was commonly achieved by nebulization or the use of novel nanomaterial formulation strategies for optimal pulmonary delivery.^31^ Nebulized, convalescent antibodies also had the benefit of dose sparing effects due to localized lung delivery.^32^ Aerosolized delivery of single-domain antibody fragments, or nanobodies, were also engineered.^33,34^ Nanobodies are advantageous since its smaller size reduces manufacturing costs. (Pittsburgh inhalable Nanobody 21) PiN-21 is an example of a successful nanobody that reduced SARS-CoV-2 infection to levels below 0.1 ng/mL in *in vitro* models and decreased lung viral titers for 6-log reduction in *in vivo* hamster models.^34^ Synthetically designed nanobodies that bind SARS-CoV-2 spike protein, prevent binding with human ACE2 receptors.^33^ Nanobodies were rationally designed using techniques such as yeast surface-displayed synthetic libraries and affinity maturation,^33^ camelid immunization and proteomics modeling.^35^ The power of these high throughput synthetic library screening, integrated modeling, facilitated the fast and accurate development of new therapeutics to target emerging virus variants.^36^

## Rapid diagnostic technology

With the rapid spread of the SARS-CoV-2 infections, there has been a rising demand for rapid and accurate testing methods. Diagnostic tests are especially important to detect cases and isolate cases to prevent the spread of infection. Diagnostic tests fall into two categories: molecular and antigen testing.^37^ Molecular testing involves detection of the viral genetic material and has largely used reverse-transcriptase real-time PCR (RT-PCR) for DNA amplification.^38^ While these tests have high accuracy, they also require specialized equipment and technicians and can also take hours for detection.^39^ Thus, rapid-antigen detection (RAD) tests (or “lateral flow tests”) have been developed that do not require specialized equipment and can output results as fast as 30 min^39^ (**Table ****II**).

**Table II:** Approved SARS-CoV-2 diagnostic rapid tests.

Product	Mechanism	Date EUA Issued/Updated	Authorized Settings*	Assay Time	References
BD Veritor System for Rapid Detection of SARS-CoV-2ʹ	Chromatographic Digital Immunoassay; read from instrument	Original: 7/2/20 Updated: 3/31/21	L, W	~ 15 min	40
BinaxNOW COVID-19 Antigen Self Test	Lateral Flow, Visual Read	Original: 3/31/21 Updated: 4/1/21	L, W, H	~ 15 min	40
Ellume COVID-19 Home Test	Lateral Flow, Fluorescence, Instrument Read	Original: 12/15/20 Updated: 2/11/21	L, W, H	~ 15 min	40
Clip COVID Rapid Antigen Test	Lateral Flow immuno-luminescent assay, Instrument Read	12/7/20	L, W	~ 30 min	40

Some EUA issued antigen diagnostic tests for SARS-CoV-2. Updated as of 4/1/21. *L, laboratories certified under clinical laboratory improvement amendments that meet requirements for high or moderate complexity tests; W, patient care settings; H, home.

The accessibility of RAD tests has allowed for the development of diagnostic kits that are widely available to the public over-the-counter and through health care providers. In fact, the first self-testing kit was granted emergency use approval (EUA) by the FDA in November 2020 and since then the number of EUA testing kits have grown to 15 as of 3/26/2021 with many others across the globe with CE-IVD.^40,41^ However, because the antigen detection with RAD is less sensitive compared to viral DNA detection by RT-PCR, a high amount of viral load is often necessary for detection, which results in varying sensitivities among different kits when viral loads are low.^42^ The continuous monitoring and effective quality control of RAD kits are essential to assess false-negatives resulting from insufficient sensitivity.^43^ Independent organizations such as the Foundation for Innovative New Diagnostics (FIND) are running surveillance on assays available throughout the globe to ensure that they have high sensitivity and specificity (standard: ≥ 80% sensitivity and ≥ 97% specificity).^41^

## Repurposing with purpose

The rapid need for therapeutics to treat moderate to severe COVID symptoms prompted the use of computational tools to speed up drug repurposing.^44^ The benefits to repurposing drugs are not new. These drugs are already FDA approved and have established manufacturing and distribution chains. Nonetheless, there are thousands of available therapeutics which have different molecular targets, varying purposes (i.e., antiviral, anti-inflammatory), as well as different drug types (i.e., antibody, small molecule, enzymatic inhibitors). To solve this challenge, computational tools have been implemented. Multiple studies were released that mapped the SARS-COV-2 protein interaction with human proteins to identify druggable proteins and existing compounds.^45^ Notably, one VINI *in silico* model found a combination of HIV drugs that could successfully inhibit SARS-CoV-2 spike glycoprotein.^46^

Many of the computational models being employed are possible because of the open-source data sets of FDA approved drugs. While there are many, a great example is the NIH NCATS developed as an OpenData Portal, a compilation of SARS-CoV-2 screening related assays against all FDA approved drugs and related assay protocols.^47^ The OpenData Portal is cited in numerous peer-reviewed papers and has successfully allowed researchers to identify SARS-CoV-2 potential drug candidates that were not but also allowed for the discovery of others that were previously unidentified.^48^ While these targets still have to be validated *in vitro* and through human clinical trials,^44^ this accelerated the decision-making process for drug discovery.

## Future outlook

The collaboration amongst science, industry, and government that occurred during COVID-19 was an enormous achievement. This presents a great opportunity to commemorate the successes and reflect on the advancements that will persist after the pandemic. The mRNA vaccine field has been reinvigorated and is now being applied to other infectious diseases.^49^ Even after the COVID-19 pandemic ends, mRNA vaccines will likely be a focus of vaccine development for prototype pathogens now that the mRNA-LNP platform is available and proven efficacious. It should be noted that the investment in technologies that had potential but whose application was not fully realized was critical preparation for the fast development of the COVID mRNA vaccines. The success of penicillin development during WW2 is just as valid of a lesson as the government support of mRNA vaccine development in 2020.

While it is easy to have a focus on the final product, the rapid enactment of COVID-19 related diagnostics and therapeutics was rooted in pre-pandemic basic research in biomaterials. Decades of lipid nanoparticle engineering^50^ preceded the success of the lipid formulated mRNA vaccine. The use of mRNA as a vaccine system depended on decades-long research into the stabilization of mRNA nucleotides.^51^ Biocatalytic reactions research fueled protein engineering and the development of rationally designed antibodies and nanobodies.^52^ Moreover, the 3D manufacturing that drove the protective equipment pandemic needs was facilitated by basic research in polymer chemistry and stereolithography.^53^

Like the development and production of penicillin circa World War II, the speed and scale of the technological advancements achieved during the COVID-19 pandemic can be attributed to the funding of both technology and basic research. The US Department of Health and Human Services and the National Institutes of Health formed Operation WARP Speed to support the manufacturing and distribution of therapeutics, diagnostics, and protective equipment.^54^ The COVID-19 scientific and technological breakthroughs have led to unprecedented increases in budgets that will impact research funding even after the pandemic. In 2020, DOE and NIH budgets increased by $USD11.25 billion.^55^ NSF funding will potentially increase by $USD100 billion over the next five years, its largest increase since its founding in 1950.^56^ Similarly, the private sector has benefited. In 1944, the government funded success of penicillin catapulted Merck, Pfizer, Abbott, and what is now Bristol Myers Squib into a new pharmaceutical era.^57^ In 2020, Moderna went from an early-stage, high-risk biotech company worth $USD6.5 billion to having a market value of $USD100 billion.^58^

This is also a time to correct issues that may have been overlooked. The rush toward repurposing suitable COVID-19 therapeutics lead to the promotion of hydroxychloroquine. Interestingly, the hydroxychloroquine literature became highly contested following a flurry of retractions in high-tier publications. Several of these publications were based on electronic health records which were readily attainable but hard to verify.^59^ In follow-up clinical trials, hydroxychloroquine had no therapeutic benefit,^60^ or in some studies, led to worse outcomes in COVID-19 patients.^61^ In addition, its use as a COVID-19 therapeutic created long-term supply shortages that affect other chronic patient populations such as individuals living with Lupus.^62^

The speed at which funding resources were dispensed have left out underrepresented minority communities. COVID-19 exacerbated preexisting health disparities^63^ and decreased the years of potential life lost (YPLL) in non-Hispanic black and Hispanic individuals.^64^ In the United States, the distribution of COVID-19 vaccines brought a reprieve to the worst conditions of the still ongoing pandemic. However, this was not the case for many countries. Limited access to vaccines and other COVID medical interventions across countries have resulted in health care and economic inequalities.^65^ As countries raced to develop their own vaccines, there was a rise in vaccine nationalism.^66^ Moreover, with wealthy nations purchasing virtually all of the available vaccines, leaves people in less resourced places little to no opportunity to become vaccinated until at least 2022.^66^

Regardless, the future is bright for multidisciplinary driven technology development. The COVID-19 pandemic has fostered a new era of convergence science, the integration of multiple disciplines and methods to solve complex problems. A broad example of this is the convergence of political, and public health systems to mount a multiscale preparedness plan for future pandemic threats.^67^ This has also been applied in the scientific realm. Convergence among nanomedicine, medical devices, and computational tools to develop pandemic preparedness pipelines.^68^ For better or worse, COVID-19 has shaped the next era of scientists, doctors, public health experts, and politicians. The rapid growth seen in the last two years will surely have impacts that last decades. It is the authors hope that science and society will be better for it.
